# Strengthening care in collaboration with people with lived experience of psychosis in Uganda (SCAPE-U): A protocol for a cluster randomized controlled feasibility trial

**DOI:** 10.1186/s40814-025-01684-8

**Published:** 2025-07-21

**Authors:** Byamah Brian Mutamba, Sauharda Rai, Lynn Semakula, David Cappo, Laura Asher, Wilfred Gwaikolo, Brandon A. Kohrt

**Affiliations:** 1 YouBelong Uganda, Kampala, Uganda; 2 Butabika National Referral Mental Hospital, Kampala, Uganda; 3 Center for Global Mental Health Equity, The George Washington University, Washington DC, USA; 4 University of Nottingham, Nottingham, UK

**Keywords:** Community health workers, Community-based participatory research, In service training, Mental health recovery, Primary health care, Psychiatric rehabilitation, Psychotic disorders

## Abstract

**Background:**

Mental health services are most effective and equitable when designed, delivered, and evaluated in collaboration with People With Lived Experience of mental health conditions (PWLE). However, PWLE are rarely involved in health systems strengthening, and when they are, it is limited to specific components (e.g., peer helpers) rather than multi-tiered collaboration in the full continuum of home to community to facility based services. Moreover, programs that do involve PWLE typically involve people with a history of substance use conditions or common mental disorders. The collaboration of People With Lived Experience of Psychosis (PWLP) is especially rare. Therefore, we aim to explore the feasibility of collaborating with PWLP for health systems strengthening in this feasibility trial.

**Methods:**

This pilot cluster randomized controlled feasibility trial will randomize 36 health facilities to a standard implementation arm where primary care workers (PCW) will be trained by mental health specialists (control), or a collaborative care model with added co-facilitation of PCW trainings by PWLP as well as home visits by PWLP to service users (intervention). The intervention condition is referred to as “Strengthening CAre in collaboration with People with lived Experience of psychosis in Uganda” (SCAPE-U). The 36 health facilities will be distributed across six clusters with three clusters in each arm. PhotoVoice will be used to train PWLP to be co-facilitators of PCW training and provide home-based support to service users in the intervention arm. The primary outcomes of the feasibility trial will be the feasibility, acceptability, and safety of collaborating with PWLP. Data will also be collected on individual-level outcomes for PCWs, and service users to inform the feasibility of data collection and obtain effect size estimates.

**Discussion:**

Findings from this feasibility trial will inform a fully powered trial to evaluate the benefits of an implementation strategy characterized by collaboration with PWLP across the continuum of healthcare services.

**Trial registration:**

ClinicalTrials.gov. number: NCT05863572.

Date of registration: May 18, 2023.

URL of trial registry record: https://clinicaltrials.gov/study/NCT05863572?term=NCT05863572&rank=1.

## Background

There is a large global burden of untreated psychosis, with scarcity of evidence-based treatment delivered in low-income countries [1]. The lifetime risk of psychosis is estimated to be 3% globally [2]. Psychosis is associated with significant impairment in all aspects of life, often resulting in social alienation, violation of human rights, and other complications such as higher rates of suicide attempts, substance abuse, and homelessness [3, 4]. Among primary psychoses, schizophrenia is the most prevalent, affecting at least 24 million people worldwide, and has the strongest association with disability [3, 5]. According to the 2019 Global Burden of Disease, schizophrenia carried the heaviest disability weight of all mental disorders. Moreover, compared to the general public, the odds of early death are nearly 3 times higher among persons living with schizophrenia [6].

Although psychosis can be highly disabling, it can be treated, and early intervention can be critical in slowing the progression of psychotic disorders to chronic or more severe stages of disease [4, 7]. Following treatment, one in three people with schizophrenia can experience total remission of symptoms [8]. Early diagnosis and intervention also limit the duration of psychotic episodes and reduce recurrence [3, 7]. Unfortunately, at least two out of three people with psychosis globally do not have access to specialist mental health care [1]. In low- and middle-income countries (LMIC) such as Uganda, more than 80% of individuals with all types of mental health conditions including psychosis have no access or limited access to any form of mental health services owing to lack of a trained mental health workforce, poor infrastructure, and pervasive stigma [9].

Uganda exemplifies low-income countries where the majority of treatment for people living with psychosis (PLWP) is restricted to a small number of psychiatric hospitals. There are almost no mental health services available at primary care and community levels in Uganda for People with Lived Experiences of Psychosis (PLWP) [10, 11]. Moreover, Uganda’s health system is inadequate to provide holistic management for PLWP [4, 9]. Pharmacological treatment is typically the sole treatment with neglect of other essential aspects of evidence-based care including psychosocial interventions, skills training, family psychoeducation, rehabilitation, and reintegration into the community following discharge from hospital [12–14]. A 2018 study conducted at Uganda’s National Referral Mental Hospital found that of 156 service users managed for first episode psychosis, 84% received antipsychotics, whereas only 2% and 13%, respectively, received a vocational plan and multifamily group psychoeducation [15].

The World Health Organization (WHO) recommends a shift from institutionalization of people suffering from mental health conditions to community-based models of care, including integration of mental health services into primary health care systems [16, 17]. The current need in LMIC is to increase availability of evidence-based treatment for PLWP including psychosocial interventions. However, the evidence base for community-based psychosocial interventions for people with psychosis in low-income countries is very limited [18].

In parallel, there have been increased calls to collaborate with People With Lived Experience of mental health conditions (PWLE) to strengthen health systems [19–21]. Collaboration with PWLE during the training of primary care workers has the potential to reduce healthcare workers’ stigma and improve their diagnostic accuracy for mental health conditions [22]. However, most research to date has focused on peer-support programs [19, 23–26], and the vast majority of studies are based in high-income countries. A Cochrane review on the use of peer support for persons with psychosis and other serious mental illnesses found that, in comparison with standard care, peer support may improve behavioral domains such as hope, agency, recovery, empowerment, and personal confidence in addition to mental state [24]. Peer support is reported to have benefits such as reduced hospitalization and better recovery outcomes for persons with serious mental illness [25, 27].

In Uganda, only two studies have looked at PWLE involvement, both using peer support. The BRAIN GAIN feasibility study showed feasibility and acceptability of the peer support intervention for people living with serious mental health conditions [28] while the UPSDIES trial, which looks at empowering mental health services through peer support, is underway [29].

Evidence for other aspects relating to the feasibility of involving people with lived experience in health systems strengthening is scarce. The Brain Gain study at Butabika hospital aimed to promote recovery among inpatient and outpatient service users [28]. Few studies globally, and no studies in Uganda, have evaluated the feasibility and acceptability of collaboration with PLWP across the continuum of services from the facility to the community to the home, with an aim of eventually assessing the impact of such initiatives.

**Fig. 1:**
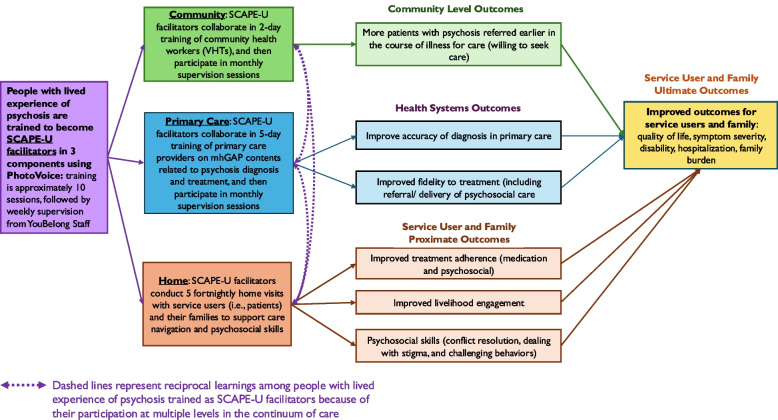
Conceptual framework of SCAPE-U

We plan to address these gaps through a strategy called “Strengthening CAre in collaboration with People with lived Experience of psychosis in Uganda” (SCAPE-U) where PWLP are engaged in co-facilitating mental health trainings of primary care workers and community health workers and providing support to families of people with psychosis currently in treatment. By using this comprehensive and continuum of care approach, our hypothesis is that engaging PWLP can improve quality of mental health care (including accuracy of diagnosis and fidelity to treatment in primary health care level), increased help seeking and referral at the community level and improved support for treatment adherence and livelihoods at the family level (Fig. 1). We further hypothesize that this model can facilitate early detection, reduce duration of untreated psychosis, overburdening of hospital services, and ultimately improve the quality of life of PWLP.

## Objectives

This current protocol describes a feasibility study using a cluster randomized controlled trial (c-RCT) design. The pilot study compares implementation as usual for mental health services to the SCAPE-U implementation strategy, in which PWLP collaborates in training primary care workers and community health workers, as well as conducting home visits to service users and their families. The pilot and feasibility study have the following objectives (see Table 1):

1. To assess the feasibility and acceptability of involving people with lived experience of psychosis in mental health trainings and home visits.
2. To determine recruitment and retention rates of SCAPE-U facilitators, service users and their caregivers, primary care workers, and community health workers.
3. To establish the acceptability and feasibility of data collection procedures.
4. To demonstrate the feasibility of trial procedures.
5. To demonstrate the ethics and safety of the study procedures.
6. To describe changes in outcome measures at individual, family, and health system levels.
7. To determine the cost of the SCAPE-U implementation strategy.

**Table 1:** Feasibility trial objectives

**Objective**	**Resear**ch questions	**Hypothesis**	**Methods**	**Participants**
**1. Feasibility and acceptability of SCAPE-U**	Is it feasible to train and involve people with lived experience of psychosis in the intervention? Do primary care workers, community health workers and caregivers of service users find it acceptable to have people with lived experience of psychosis as co-facilitators in their trainings/supervision and home visits?	People with lived experience of psychosis can be trained to deliver training and home visits effectively and safely All the key stakeholders of the intervention will find the involvement of people with lived experience acceptable	Qualitative interviews Process notes Adverse event logs (target: fewer than 20% among recruited PWLE)	SCAPE-U facilitators, primary care workers, community health workers, service users and their families, research staff and trainers
**2. Recruitment and retention**	Can we recruit, train, and retain enough PLWP to be SCAPE-U facilitators? Can we recruit and retain sufficient primary care workers, community health workers and service users and their caregivers for outcome assessment?	PLWP can be adequately recruited, trained, and retained for delivering the SCAPE-U intervention For primary care workers, community health workers and service users and their families, maximum recruitment might be required to address loss to follow-up and drop-out	Process output: number of SCAPE-U facilitator recruited, completed training, and retained. Target = 50% retention For primary care workers, community health workers: number in health posts, number completing the training, supervision and completing outcome measures. Target for both = 60% at 8 months follow-up For SU and families: 50% completing the 5 session home visits	SCAPE-U facilitators, primary care workers, community health workers, service users and their families, research staffs
**3. Acceptability and feasibility measures and data collection procedures**	Are the quantitative and qualitative assessment measures and collection procedure acceptable and feasible to administer with the stakeholders at planned intervals?	The selected measures will be acceptable and feasible for measuring the intended outputs of the study	Tool completion rate, time, missingness of data (target: fewer than 20%)	Primary care workers, community health workers, service users and families, research staff
**4. Randomization**	Is cRCT feasible to run the study and measure the intended outcomes—in terms of contamination, fidelity, and implementation strategy?	Cluster randomization is feasible to execute the study and measure intended outcomes	Baseline summary of two arms Demographic details of primary care workers, community health workers (age, gender, years of experience, qualifications, education) Record on participant’s arm crossover (target: less than 30%)	Primary care workers, community health workers, service users and families, research staff
**5. Ethics and safety**	Can SCAPE-U be delivered with best ethical practice and safety of SCAPE-U facilitators, primary care workers, community health workers, service users and their families? Are there any unintended harms and how can they be addressed?	SCAPE-U intervention can be delivered with minimum harm to all stakeholders	Qualitative interviews Adverse event log Process notes	SCAPE-U facilitators, primary care workers, community health workers, service users and families, research staffs
**6. Change in outcome measures**	Do the outcome measures of the stakeholders improve after participating in the intervention?	Stakeholders in the SCAPE-U intervention arm will exhibit better outcomes than in control arm	Outcome measures assessment	Primary care workers, community health workers, service users and families
**7. Cost of intervention**	Can we measure the cost of SCAPE-U using measures and procedures from this study?	Costing of the intervention can be done in subsequent trial using procedures and measures tested in this pilot study	Cost-related outcome measures	YouBelong team, primary care workers, community health workers, service users and families

## Methods

### Setting

The study will be conducted within primary health facilities, community settings, and home settings in two urban and peri-urban districts of Kampala and Wakiso located in central Uganda, with district populations of 1,507,080 and 1,997,418, respectively [30]. Uganda’s formal health infrastructure consists of a tiered system of health centers based on the catchment area and services provided. At the lowest level, communities have village health teams consisting of volunteer community health workers tasked with coordinating health promotion activities and linking service users to care. Mental health services are meant to be provided at primary care centers (health centers III and IV) and hospitals (General, Regional and National Referral Hospitals). Services are provided by mental health professionals or general health workers depending on the tier. However, even though the integration of mental health services is part of national policy frameworks, mental health services are not well integrated into the primary health care system and are not routinely available at most health centers [31, 32]. Specialist mental health care is only accessible at two of the five national referral hospitals in the country and at 13 of 16 regional referral hospitals.

The study will be implemented by YouBelong Uganda, a Ugandan non-governmental organization involved in research, advocacy, and services for people with severe mental illness [33, 34], in coordination with the national health system and supported by the national referral psychiatric hospital at Butabika.

### Study design

The design is a cluster-randomized controlled feasibility trial comparing the standard implementation arm, where PCWs will be trained by mental health specialists, versus the intervention arm, where, in addition, PWLP will cofacilitate the PCWs training and provide home visits to the service users with psychosis.

The unit of randomization is a geographic cluster of primary care facilities. There are 36 primary care facilities in the two study districts. These will be divided into six geographic clusters based on a health subdistrict, each consisting of six primary care facilities. Three geographic clusters will be randomly allocated to the control arm and three to the SCAPE-U arm. We chose a cluster design because the interventions are delivered at a primary care facility and community level, meaning individual randomization is not possible. Having multiple health workers and primary care facilities in each cluster also ensures that we will not lose the entire cluster in the scenario that primary care workers or community health workers retire or are relocated by the government.

### Study participants

This study will involve six categories of study participants:

(i) People with lived experience of psychosis trained as SCAPE-U facilitators—8 to 10 people with lived experience of psychosis who have previously received services from YouBelong Uganda [33, 35] will be recruited. The inclusion criteria are (1) diagnosis of a psychotic disorder confirmed by a mental health professional (psychiatric clinical officer or psychiatrist). (2) Capacity to provide informed consent. The main exclusion criterion is any health, functional, or cognitive impairment, assessed through mental state examination, that could jeopardize their full and safe participation in SCAPE-U activities. There are no inclusion/exclusion criteria relating to current engagement in treatment.
(ii) Primary care workers—two primary care workers will be selected per health facility, with a target of 36 primary care workers per arm (72 total for the study). There are no exclusion criteria.
(iii) Community health workers—we will select up to five community health workers from each health facility. This will equate to approximately 90 community health workers per arm and an estimated total of 180 community health workers for the study.
(iv) Psychiatrists, psychiatric clinical officers,and psychiatric nurses—1 psychiatrist, 3 psychiatric clinical officers, and 2 psychiatric nurses who are involved in delivering training to primary care workers will be included.
(v) Service users diagnosed with psychosis by primary care workers—the primary intended beneficiaries of study interventions are service users receiving treatment for psychosis at primary care facilities. The goal is to have 10 service users per cluster, 30 per arm, and 60 in total for the study. *Inclusion criteria*: (1) persons diagnosed with psychosis at a primary health care facility in Kampala/Wakiso District, following mhGAP training as part of the SCAPE-U trial; (2) ability of the service users to consent to study enrollment and procedures, or, if the service user does not have capacity to consent, a responsible surrogate can give consent; (3) persons eligible for outpatient management of psychosis. *Exclusion criteria*: (1) persons diagnosed with psychosis requiring inpatient management; and (2) persons for whom consent for participation in the study cannot be obtained.
(vi) Family members of service users—at least one primary carer for study service users will be identified to collect outcome data from a family perspective (*n* = 30 per arm, 60 for the total study).

### Recruitment

Potential SCAPE-U facilitators will be identified and contacted by the YouBelong team. Potential service users’ participants will be identified by primary care workers working in participating health facilities. Their identification will commence after training of primary care and community health workers is complete. Following diagnosis of a service user with psychosis, as part of their usual practice, the primary care worker will contact the research assistant by telephone. The research assistant will either immediately attend the facility or arrange an alternative time to meet with the service user. Eligibility of service users will be assessed by research assistants, who will be psychiatric clinical officers by training. The research assistant will complete a screening assessment, including assessment of decision-making capacity using the University of California, San Diego Brief Assessment of Capacity to Consent (UBACC) [36]. Eligible potential participants will be given information about the study. Informed consent will be obtained for participants who are eligible and interested in participating. For all consenting participants, detailed contact information will be recorded.

### Sample size

We envision that the study sample size (60 service users and 60 linked family members) is sufficient to achieve our primary objectives, that is to assess SCAPE-U intervention acceptability and feasibility, as well as the trial and data collection procedures. This is a pilot cRCT and therefore we will not conduct a power calculation or formal hypothesis testing. Consistent with expert guidance on pilot trials, including the CONSORT extension for pilot and feasibility studies, this study is not powered to detect statistically significant between-arm differences [37]. Using an assumption of 80% participation rate, the sample size of 60 service users will enable the proportion of service users and carers who complete baseline assessments to be estimated with a 95% confidence interval width of 68.2% to 88.6%, using a Wilson score interval, which is appropriate given the small *n*. Estimates obtained in this feasibility trial will be used to inform the sample size determination for a fully powered RCT comparing the clinical and cost effectiveness of treatment as usual versus the SCAPE-U intervention.

## Interventions

### Standard training and mental healthcare implementation (control arm)

#### Primary care level

Primary care workers will be trained to screen, assess, diagnose, and treat psychosis and related disorders using the WHO mental health Gap Action Programme Intervention Guide (mhGAP-IG, version 2.0) [38]. The mhGAP-IG has been used as a package for providing mental health care in LMIC settings including Uganda [39]. This 5-day training, coordinated by YouBelong Uganda in collaboration with Butabika National Mental Hospital and the Ministry of Health in Uganda, will be delivered by psychiatrists and/or psychiatric clinical officers trained as mhGAP-IG trainers. Training will be delivered primarily in English. After the training, the primary care workers will receive monthly supervision from a psychiatric clinical officer or psychiatric nurse. This is comparable to the standard procedures used nationally for training primary care workers in mental health.

### Community level

At the community level, community health workers will receive a 2-day training based on the WHO mhGAP Community Toolkit Community Provider manual [40] to increase knowledge and skills required to identify symptoms of psychosis and other mental health problems. Additionally, they will be trained to disseminate information on psychosis at community awareness-raising meetings**.** The training will be delivered in Luganda, the predominant local language, by the trained primary care workers and experienced trainers from the YouBelong Uganda team in collaboration with Butabika Hospital. Community health workers will also receive monthly supervision by trained primary care workers after the training.

### SCAPE-U (intervention arm)

The initial SCAPE-U intervention was developed through an 18-month contextualisation phase, consisting of theory of change workshops, a single-arm small-scale pilot study (involving one cluster, 9 SCAPE-U facilitators and 10 service users) and qualitative interviews. The final SCAPE-U intervention to be tested in the feasibility trial was designed based on findings from the contextualisation phase.

Figure 1 provides an overview of the participation of SCAPE-U facilitators across the tiers of primary care, community, and home-based care. SCAPE-U facilitators will receive a travel compensation for attending facilitator/PhotoVoice training, health worker training, and home visits. However, they will not receive a salary.

### SCAPE-U facilitator training

The training will prepare selected people with lived experience of psychosis, and the YouBelong community recovery program, to participate in primary care and community health worker training and to conduct home visits; they will be trained in PhotoVoice and hereafter referred to as SCAPE-U facilitators. PhotoVoice is based on participatory methods where photography is a medium of storytelling and message delivery [41–43]. SCAPE-U facilitators will use PhotoVoice narratives to cofacilitate training of PHWs and CHWs, as well as provide psychosocial support during home visits. The training, which will be delivered in Luganda, will involve a 2-day residential, followed by eight weekly sessions. SCAPE-U facilitators will learn about using cameras and taking pictures, writing recovery stories, practicing storytelling using photographs, public speaking, advocacy, addressing stigma, and managing confidentiality and disclosure. PhotoVoice has successfully been used to reduce stigma among persons living with severe mental health conditions and to facilitate peer support and community reintegration in the USA [44, 45]. The approach has also been tested through a pilot study in Nepal where people with lived experience of depression, psychosis, and alcohol use disorder participated in mhGAP-IG trainings of primary care workers [46, 47]. Findings from this study suggested this approach to training improved diagnostic accuracy and decreased stigma amongst primary care workers [22]. Details of the PhotoVoice training are described elsewhere [43].

### Primary care level

Primary care workers and community health workers will receive training with the same duration and broad content as the standard implementation (control) arm. However, in the intervention arm, SCAPE-U facilitators will co-facilitate trainings alongside mental health professionals. During the primary care worker training, SCAPE-U facilitators will present sessions on living with psychosis, using their PhotoVoice stories. SCAPE-U facilitators will describe the symptoms of psychosis from an experiential perspective, specifically the experience of suffering, negotiating pathways of care and treatment, including an emphasis on the benefit of psychosocial support in addition to medication. SCAPE-U facilitators will also conduct question and answer sessions so that primary care workers can ask them about their experiences. In addition, SCAPE-U facilitators will be present during the other training sessions so that primary care workers can interact with them in both structured and unstructured social activities. At the conclusion of the training, SCAPE-U facilitators will hold a session on anticipated challenges of primary care workers, and with the primary care workers, they will collaboratively come up with solutions to address these challenges. SCAPE-U facilitators will also collaborate in the supervision of primary care and community health workers after their trainings are completed.

### Community level

In addition to standard training, community health workers in the intervention arm will receive an added component in which SCAPE-U facilitators present recovery narratives, aimed at improving the accuracy of identifying individuals with possible psychosis within their communities. The narratives will incorporate elements of early detection, referral, and care, along with stigma reduction. The aim of the SCAPE-U facilitators’ participation is to motivate community health workers to strengthen the linkage and referral between community and primary healthcare services. SCAPE-U facilitators will also participate in supervision sessions with the community health workers after their training to help raise issues about the community and other aspects of the care pathway.

### Home level

Service users receiving service from the health facilities in the control arm will not receive any additional service besides that provided from health facilities. For those receiving service from the intervention arm and diagnosed with psychosis, SCAPE-U facilitators will conduct five 1-h home visits to service users who have been diagnosed with psychosis by primary care workers and are currently receiving treatment. Primary caregivers to service users are expected to be present during these visits. Each session will consist of SCAPE-U facilitators sharing their lived experiences through a 10-min presentation of an aspect of their PhotoVoice story. Each session will be tailored to a psychosocial theme (medication adherence, conflict resolution, stigma, livelihood support, and dealing with challenging behaviors) [33, 35]. There will then be time for questions and discussion with the service users and their family (Table 2).

**Table 2:** Overview of services and implementation by study arm and level of care

Care level	Standard services arm	SCAPE-U arm
Primary care	mhGAP-IG training conducted by a psychiatrist and/or psychiatric clinical officer for 5 days with a focus on psychosis diagnosis and treatment	mhGAP-IG training conducted by a psychiatrist or psychiatric clinical officer combined with a SCAPE-U facilitator (people with lived experience of psychosis) presenting recovery stories and other activities
Community	2-day training conducted by trained psychiatric clinical officer or psychiatric nurse on identification and referrals of people potentially experiencing psychosis	2-day training conducted by trained psychiatric clinical officer or psychiatric nurse, combined with SCAPE-U facilitator presenting recovery stories and other activities
Home	No services	5 fortnightly sessions of psychosocial support provided by SCAPE-U facilitators using PhotoVoice narratives based on themes in the YouBelongHOME intervention

### Supervision of SCAPE-U facilitators

Supervision meetings with SCAPE-U facilitators will involve both technical supervision (relating to sharing of stories using PhotoVoice during training and home visits) and supportive supervision, which will focus on addressing any emotional, social, or economic challenges faced by the facilitators. The supervisors will be members of the YouBelong team. Once a month, each SCAPE-U facilitator will receive 1 h of group supervision and 30 min of individual supervision at the health facility. This will be an opportunity for the SCAPE-U facilitators to provide feedback from their experiences working with health workers and conducting home visits. This will provide a feedback channel to the primary care workers about these other aspects of the care system.

### Randomization and blinding

Randomization of clusters in the feasibility trial will be completed prior to participant recruitment and will be conducted by an independent statistician using Stata. Covariate-constrained clusterrandomisation will be used to assign health facility clusters to study arms while ensuring balance across key variables {Hemming, 2023 #120}[48]. Covariate-constrained randomization is a form of restricted randomization; specifically, the randomization will be constrained on two characteristics: number of health facilities per arm, and rural vs. urban location.

Constrained randomisation helps prevent baseline imbalances that can occur by chance, particularly in studies with a limited number of clusters, such as feasibility trials [48]. For logistical reasons, research assistants/data collectors will not be blinded. PCWs and CHWs will be blinded to the arm allocation. We will have separate mhGAP-IG trainers for each arm, and these will be blinded. Because SCAPE-U facilitators will be engaged only in the intervention arm, and only service users accessing care at PHCs in the intervention arm will receive the home visit intervention, they will both be blinded by the cluster randomized nature of the trial.

### Measures/outcomes for feasibility criteria and other objectives

The primary outcome of this trial is to understand the feasibility and acceptability of the SCAPE-U intervention. We have developed a set of quantitative and qualitative measures to do this as guided by recommendations from pilot study reporting [49]. These measures will determine what components of the SCAPE-U intervention will be included, excluded, or modified in the future full trial. The following quantitative criteria have been set to determine if this feasibility trial can be scaled up for a fully powered trial.

- Recruitment: at least 2 primary care workers were recruited in the study from each of the 36 health facilities selected.
- Retention: 50% of SCAPE-U facilitators retained by the end of PhotoVoice training, 60% of primary care workers, 60% of community health workers retained in post until the end of the 8 months follow-up period, and 50% of service users and families retained by the end of 5 session home visits.
- Contamination: less than 20% of cross-arm movement of primary care workers, community health workers, service users, and their families during the 8-month follow-up period.
- Cluster comparison: measuring baseline characteristics of recruited primary care and community health workers across both arms. We will collect information on primary care workers and community health workers’ education qualifications, gender, prior mental health training, age, and years of experience and determine balance between arms.
- Data collection procedure: less than 20% of missingness in collected data.
- Safety: fewer than 20% of adverse events among recruited SCAPE-U facilitators and service users.

Our qualitative criteria for this feasibility study are presented in Table 3.

**Table 3:** Assessment of feasibility and acceptability of the intervention and trial procedures

Domains	Research questions	Methods
Feasibility and acceptability of SCAPE-U intervention	Do psychiatrist/psychiatric clinical officer, primary care workers, and mental health service users find it feasible and acceptable for PhotoVoice trained people with lived experience to participate as co-facilitators in training and supervision?	● Qualitative interviews with psychiatrist/psychiatric clinical officer, primary care workers, SCAPE-U facilitators, and research staff ● Process notes from trainings and supervision sessions
Fidelity and contagion of intervention	Can fidelity be feasibly and reliably assessed using a fidelity checklist? What degree of fidelity to SCAPE-U is achievable? Can contamination be captured through fidelity and other assessments?	● Qualitative interviews with psychiatrist/psychiatric clinical officer trainers, primary care trainees, SCAPE-U facilitators, and research staff ● Process notes and fidelity checklist from trainings and supervision sessions ● Logs of health workers and service users’ movement across arms
Ethics and safety of trial	Does the research pose harm to primary care workers, service users, or SCAPE-U facilitators, and are these harms adequately prevented, documented, and addressed?	● Qualitative interviews ● Service Users Collaboration Checklist ● Process evaluation notes ● Monitoring of adverse events

*Qualitative data* will be collected from PWLP, primary care and community health workers, service users, and their families, mental health specialists involved in training and research staff involved in data collection through key informant interviews (KIIs) and focus group discussions (FGD). We will conduct KIIs with 75% of the PWLP recruited to be SCAPE-U facilitators and who have completed PhotoVoice training and co-facilitated health workers training and home visits during two timepoints—after the health workers training and after home visits. For those who are just part of the PhotoVoice training, we will conduct an FGD talking about their experiences with PhotoVoice. For PCWs and VHTs, we will conduct KIIs with 15% of the total trained population. Selection will be based on gender, performance based on supervision notes, the number of mental health cases attended, and the level of health facility. We will interview them at 3 months post-training, i.e., during the first supervision. Twenty percent of the service users and their families receiving home visits will be interviewed after the completion of all five sessions. Finally, during the study’s end phase, we will interview all the trainers in both arms and research assistants involved in SCAPE-U data collection.

KIIs and FGDs will be conducted either in Luganda or English based on the participant’s preference and will be audio recorded. KIIs and FGDs will be held in either health facilities, YBU office, service user’s home, or anyplace deemed safe and confidential by both the interviewer and interviewee and will be conducted by YBU staff trained and with prior experience in qualitative data collection. All participants will be provided with information about the purpose of the KII/FGD and have written informed consent taken by the researcher.

*Quantitative data* will be collected to address objectives 3 and 6 (Table 1) of this feasibility trial and are related to quantitative outcomes listed in Table 4. Service user symptom severity will be assessed using the Positive and Negative Symptoms of Schizophrenia (PANSS) scale [50]. This tool measures the severity of positive and negative symptoms, with higher scores equating to greater symptom severity and has been widely used in psychosis research trials. Additional outcomes for the service users include quality of life measured using the World Health Organisation Quality of Life Brief Version (WHOQOL-BREF) [51] and European Quality of Life Five Dimension questionnaire (EQ5D-5L) [52], stigma measured through the Discrimination and Stigma Scale short version (DISCUS)[53], social inclusion measured through the Social Inclusion Scale (SIS) [54], and the number of hospitalization episodes coming from the service users’ self-reporting (see Table 4). A separate costing outcome will be included for the training of service users and primary care workers and health system context regarding cost for receiving and delivering care measured through the Client Service Receipt Inventory (CSRI) [55]. Impact on family caregivers will be measured through the Family Interview Schedule—Impact on Caregivers (FIS-IC) [56].

**Table 4:** Quantitative outcome measures

Outcomes	Instrument or data collection strategy	Type of data	Respondent or source	Assessment period
PWLP recruited to become SCAPE-U facilitator				
Quality of Life	World Health Organization Quality of Life-Brief version (WHOQOL-BREF)	Self-report scale	PWLP as SCAPE- U facilitators	Enrolment, baseline,
Unintended consequences of participation in SCAPEU	Service Users Collaboration Checklist (SUCC)	Self-report scale guided with probing questions	PWLP as SCAPE- U facilitators	Mid and end point of PhotoVoice training, end of health workers training and PhotoVoice
Symptoms of psychosis	Positive and Negative Symptoms of Schizophrenia (PANSS) scale	Adapted scale for self-report	PWLP as SCAPE- U facilitators	
Service users with psychosis diagnosed by primary care workers				
Symptoms of psychosis	Positive and Negative Symptoms of Schizophrenia (PANSS) scale	Adapted scale for self-report (or report by a family member)	Service users or family members	Baseline, 4 and 8 months follow-up
Quality of life	World Health Organization Quality of Life-Brief version (WHOQOL-BREF)	Self-report scale	Service users	Baseline, 4 and 8 months follow-up
Quality of life (for health economics analyses)	EuroQoL 5-Dimension 5-Level (EQ-5D-5L)	Self-report scale	Service users	Baseline, 4 and 8 months follow-up
Stigma experienced by persons living with mental illness	Discrimination and Stigma Scale-Brief version (DISCUS)	Self-report scale	Service users	Baseline, 4 and 8 months follow-up
Social inclusion	Social Inclusion Scale (SIS)	Self-report scale	Service users	Baseline, 4 and 8 months follow-up
Hospitalization	# days hospitalized during study period	Self-report, family report, and/or hospital records	Service users or family members	4 and 8 months follow-up
Costs of services received	Client Service Receipt Inventory (CSRI)(54)	Self-report and/or family report	Service users or family members	4 and 8 months follow-up
Family members and caregivers of service users				
Impact on family members	Family Interview Schedule—Impact on Caregivers (FIS-IC) (56)	Family report	Main caregiver	Baseline, 4 and 8 months follow-up
Community health workers				
Attitudes towards people with psychosis	Social Distance Scale (SDS)	Self-report scale	Community health worker	Pre- and post-training
Accuracy of psychosis detection	Community health worker referral tool	Percent of community health worker referred patients who meet SCID criteria	Community health worker	Post training
# of individuals referred	Community health worker referral tool	Numbers	Community health worker	Throughout study
# of individuals referred by CHWs who receive a diagnosis of psychosis from PCP	Health facility records	Numbers	Research assistant reviewing clinical records	Throughout study
Primary care workers				
Outcomes	Instrument or data collection strategy	Type of data	Respondent or source	Assessment period
Attitudes towards people with psychosis	Social Distance Scale (SDS)	Self-report scale	Primary care workers	Pre- and post-training, and at final supervision session
Knowledge of psychosis diagnosis and treatment	mhGAP Knowledge	Self-report knowledge test	Primary care workers	Pre- and post-training, and at final supervision session
Clinical diagnosis skills in structured role plays	ENACT	Structured observational rating scale	Trained raters	Pre- and post-training, and at final supervision session
# of individuals diagnosed with psychosis	Health facility records	Medical records	Research assistant reviewing clinical records	Pre- and post-training, and at final supervision session
Fidelity of treatment to mhGAP guidelines	Fidelity Checklist	Observer completed fidelity checklist	Observations	Pre- and post-training, and at final supervision session
Accuracy of diagnosis (confirmatory diagnosis with SCID by PCO)	DSM-V psychosis module (SCID)	Structured clinical interview	Psychiatric clinical officer	Pre- and post-training, and at final supervision session

For primary care workers, the outcomes include attitudes measured through the Social Distance Scale (SDS) [57], knowledge measured through the mhGAP Knowledge assessment [58, 59], clinical competency measured through ENACT [60], fidelity of treatment measured through the fidelity checklist, and number of diagnosis data coming from health facility records. To determine diagnostic accuracy, a psychiatric clinical officer will use the SCID [61] to determine the diagnosis of a subset of service users diagnosed by the primary care workers. The psychiatric clinical officer’s diagnosis will be taken as the gold standard and matched with the primary care workers’ diagnosis.

Community health worker outcomes will include attitudes towards people with mental health conditions measured through the Social Distance Scale (SDS) [57] and data on number of referrals to primary care, and accuracy of psychosis detection derived using health facility data.

Timelines for quantitative data collection are presented in Table 4. Data will be collected by research assistants at the training site, health facility, or at the participants’ home. Study data will be collected and managed on Android tablets using REDCap (Research Electronic Data Capture) electronic data capture tools hosted at Infectious Disease Institute, Uganda. REDCap is a secure, web-based software platform designed to support data capture for research studies, providing (1) an intuitive interface for validated data capture; (2) audit trails for tracking data manipulation and export procedures; (3) automated export procedures for seamless data downloads to common statistical packages; and (4) procedures for data integration and interoperability with external sources [62]. Participants will receive 10,000 Ugandan shillings (US$ 2.70) for each completed assessment to compensate for time and travel costs (Table 5).

**Table 5:**
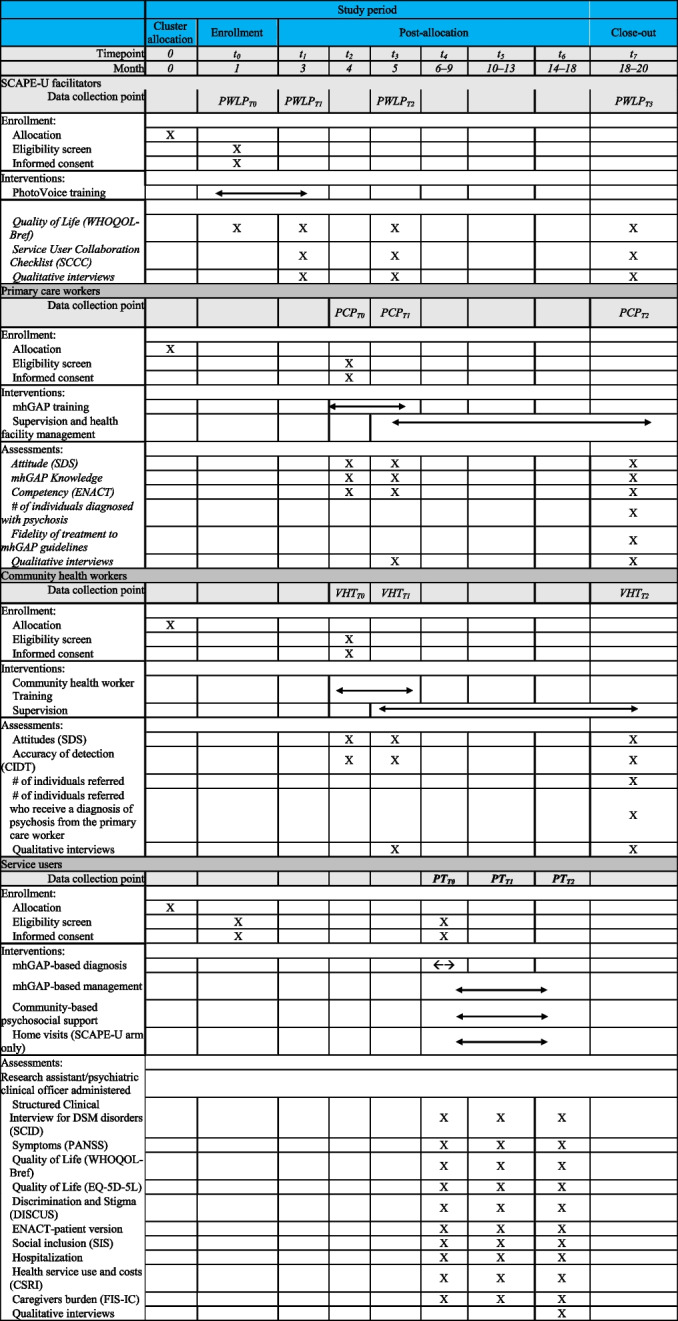
Participant timeline

*t0* pre-[LE1] PhotoVoice, *t1 *post-[LE2] PhotoVoice, *t2*[LE3] mhGAP/community health worker pre-training, *t3*[LE4] mhGAP/community health worker post-training,* t4[LE5] * service user enrollment, *t5*[LE6] 4-month post-enrollment,* t6*[LE7] 8-month post-enrollment *t7*[LE8] study close out

## Data analysis

### StataCorp LLC

Quantitative data will be exported to Stata 18 for statistical analysis where it will be analyzed per objective [63]. Data will be presented using frequency tables and figures. For continuous outcomes we will report means and standard deviations (or medians and with interquartile ranges, where data are skewed) and for categorical outcomes we will report raw counts (number, %) by treatment arm and overall. We will estimate intra-cluster correlation coefficients to inform the design of a subsequent fully powered trial.

We will calculate descriptive statistics (mean, standard deviations) for all quantitative outcomes, and these will be used to conduct power analyses for a subsequent definitive trial. Baseline data will be explored for comparability between treatment arms to guide future randomization and recruitment strategies.

Change in psychosis symptoms as a secondary outcome will be measured using PANSS scores summarized using means (SD) or medians (IQR), as appropriate based on distribution, at baseline, month 4, and month 8 within each arm. Mean and percentage changes from baseline to month 8, along with corresponding between-arm differences, will be presented with 95% confidence intervals to assess precision and potential effect size. Estimates will be obtained using analysis of covariance (ANCOVA) and mixed-effects linear regression, with health facility modeled as a random effect to account for clustering. Where PANSS scores are skewed, log transformation will be applied prior to analysis. Change analyses will be restricted to participants with at least one post-baseline assessment (modified intention-to-treat population).

### Qualitative data

(FGDs, KIIs, and process evaluation notes will be coded in Dedoose [64] and analyzed using content analysis for themes on feasibility, safety and acceptability. Themes on feasibility will be informed by the experience of implementing various study activities. In addition, other themes will relate to the experience of service users as trainers, relevance to clinical care, training duration, structure of training, content of training, and follow-up engagement. Coding will be done by multiple independent raters, and inter-rater reliability will be calculated using Kappa scores.

### Trial oversight

A Data Safety and Monitoring Board will be established to provide oversight and will include psychiatrists, epidemiologists, public health specialists, and mental health advocates, who will be independent of the study team. A DSMB protocol will be developed to guide the study team. The DSMB’s role will include monitoring recruitment progress, adverse events, deaths related to study participation, and protocol violation. All of this will be guided by dedicated standard operating procedures and will include reporting to the DSMB and ethics committees.

## Ethics

### Informed consent

Service users who meet the eligibility criteria will have the purpose of the research and study procedures, including risks and benefits, explained to them or their caregivers by use of information sheets in Luganda. Trained research assistants will only subject the selected individuals to study tools after obtaining written informed consent. No participant will be denied any treatment that they should receive, irrespective of whether they consent to participate in the trial or not. Service users identified with health problems at a severity above the level of care available will be referred to the next higher level health facility for proper management.

Written consent will also be taken from primary care workers, community health workers, and SCAPE-U facilitators prior to data collection.

### Possible harm and safety

There is a possibility of psychological distress amongst the SCAPE-U facilitators during the PhotoVoice training, and whilst conveying their stories during the health worker training or home visits, as it involves recalling things that might be unpleasant. However, considering similar experiences in Nepal, we anticipate minimum and manageable distress [43]. To ensure the safety of SCAPE-U facilitators, a trained mental health practitioner (counselor, social work, mental health nurse) will be present and available throughout the training. SCAPE-U facilitator training will also include sessions on distress management and self-care. YouBelong staff will also administer Service Users Collaboration Checklist during mid and end points of PhotoVoice training and after their participation in health workers training and home visits to identify any unintended consequences occurring because of their participation in the study. Each SCAPE-U facilitator will also be supported by a designated YouBelong Uganda team member to ensure continued recovery and wellness. In cases where advanced specialized care is required, we will establish a referral mechanism to the national mental hospital. The YBU team will also be monitoring their interaction with study participants (service users) to ensure there is no harm to either party. If at any point, a SCAPE-U facilitator or the service user decides to opt out of the study, they will be allowed to do so without any liabilities. All the service user participants will be receiving medication and basic psychosocial intervention by the primary care worker and are expected to receive an optimum package of available care. The primary care workers will be supervised by the psychiatrist and psychiatric clinical officer.

All study staff and procedures will be governed by the YouBelong Uganda Safeguarding policy to ensure a safe environment for study beneficiaries and SCAPE-U facilitators. Study staff and SCAPE-U facilitators will receive 1 h of training on adherence to the policy, which covers sexual abuse, emotional abuse, physical abuse, and financial abuse. Any staff or SCAPE-U facilitator with a history of child abuse will be excluded from the study. Weekly supervision meetings for SCAPE-U facilitators by YouBelong Uganda team members will ensure regular review and update of the safeguarding policy from the perspective of SCAPE-U facilitators and monitoring of the safeguarding culture during the study implementation. In addition, all project staff will undertake Good Clinical Practice and Human Subjects Protection training prior to commencement of the project.

### Dissemination

Findings from the SCAPE-U study will be presented at two dissemination meetings in Uganda, one involving key government and non-government actors, and another to be held in the study communities. Study findings will also be published in academic journals. Authorship eligibility will comply with guidelines of the International Committee of Medical Journal Editors, with additional attention to recommendations for equitable representation of researchers from LMIC for academic authorship [65]. The study materials for training and implementation of SCAPE-U will be made available through the Mental Health Innovation Network (www.mhinnovation.net). In keeping with NIMH recommendations, data will be made publicly available after publication of primary analyses. A final study report will be shared with the funder.

## Discussion

The results of this mixed-methods feasibility trial will be used in understanding the feasibility and acceptability of SCAPE-U from the perspective of a range of providers and beneficiaries and will ultimately help us in designing a similar full trial in Uganda. If we identify any issues regarding our procedures in terms of participant recruitment, retention, safety, fidelity, cost effectiveness, and data collection and management, we will modify accordingly. In case significant modifications are needed, we will consider an internal pilot in the context of the full trial.

The SCAPE-U study aims to improve access to evidence-based care for individuals affected by psychosis and their families, who live in areas with limited community mental health services. This study has the potential to significantly impact mental health care in Uganda and other LMICs. A key strength of the SCAPE-U study is its collaboration with people with lived experience of psychosis to strengthen health systems for the delivery of comprehensive mental health care from the home to the health facility. This study will provide evidence for the safety and feasibility of such collaboration to strengthen the continuum of care within the local health system.

## Data Availability

Data sharing is not applicable to this article as no datasets were generated or analyzed during the current study.

## Abbreviations

SCAPE-U: Strengthening Care in Collaboration with People with Lived Experience of Psychosis in Uganda
SCID: Structured Clinical Interview for DSM Disorders
LMICs: Low- to middle-income countries
PWLE: People with lived experience of mental health conditions
PWLP: People with lived experience of psychosis
RCT: Randomized controlled trial
HC: Health center
VHT: Village Health Teams
PHC: Primary Health Care
YBU: YouBelong Uganda
YBH: YouBelong Home
mhGAP: Mental Health Gap Action Programme
GWU: George Washington University
WHO: World Health Organization
SMI: Severe mental illness
RA: Research Assistants
FGD: Focus Group Discussion
TAU: Training as Usual
GCP: Good Clinical Practice
HSP: Human Subjects Protection
